# Catalase-deficient mice induce aging faster through lysosomal dysfunction

**DOI:** 10.1186/s12964-022-00969-2

**Published:** 2022-12-06

**Authors:** Raghbendra Kumar Dutta, Joon No Lee, Yunash Maharjan, Channy Park, Seong-Kyu Choe, Ye-Shih Ho, Hyug Moo Kwon, Raekil Park

**Affiliations:** 1 Department of Biomedical Science and Engineering, Institute of AI-Medical Science, GRI, Gwangju Institute of Science and Technology, Gwangju, 61005 Republic of Korea; 2 Department of Microbiology and Center for Metabolic Function Regulation, Wonkwang University School of Medicine, Iksan, Jeonbuk 54538 Republic of Korea; 3 Institute of Environmental Health Sciences and Department of Biochemistry and Molecular Biology, Wayne State University, Detroit, MI USA; 4 School of Life Sciences, Ulsan National Institute of Science and Technology, Ulsan, Republic of Korea

**Keywords:** Catalase, ROS, Aging, Lysosome, mTORC1

## Abstract

**Background:**

Lysosomes are a central hub for cellular metabolism and are involved in the regulation of cell homeostasis through the degradation or recycling of unwanted or dysfunctional organelles through the autophagy pathway. Catalase, a peroxisomal enzyme, plays an important role in cellular antioxidant defense by decomposing hydrogen peroxide into water and oxygen. In accordance with pleiotropic significance, both impaired lysosomes and catalase have been linked to many age-related pathologies with a decline in lifespan. Aging is characterized by progressive accumulation of macromolecular damage and the production of high levels of reactive oxygen species. Although lysosomes degrade the most long-lived proteins and organelles via the autophagic pathway, the role of lysosomes and their effect on catalase during aging is not known. The present study investigated the role of catalase and lysosomal function in catalase-knockout (KO) mice.

**Methods:**

We performed experiments on WT and catalase KO younger (9 weeks) and mature adult (53 weeks) male mice and Mouse embryonic fibroblasts isolated from WT and KO mice from E13.5 embryos as in vivo and in ex-vivo respectively. Mouse phenotyping studies were performed with controls, and a minimum of two independent experiments were performed with more than five mice in each group.

**Results:**

We found that at the age of 53 weeks (mature adult), catalase-KO mice exhibited an aging phenotype faster than wild-type (WT) mice. We also found that mature adult catalase-KO mice induced leaky lysosome by progressive accumulation of lysosomal content, such as cathespin D, into the cytosol. Leaky lysosomes inhibited autophagosome formation and triggered impaired autophagy. The dysregulation of autophagy triggered mTORC1 (mechanistic target of rapamycin complex 1) activation. However, the antioxidant N-acetyl-L-cysteine and mTORC1 inhibitor rapamycin rescued leaky lysosomes and aging phenotypes in catalase-deficient mature adult mice.

**Conclusions:**

This study unveils the new role of catalase and its role in lysosomal function during aging.

**Video abstract**

**Supplementary Information:**

The online version contains supplementary material available at 10.1186/s12964-022-00969-2.

## Introduction

Peroxisome generates several H_2_O_2_-producing oxidase that can penetrate the peroxisomal membrane and act as cellular signaling molecules. Antioxidant catalase is used to detoxify those powerful oxidants [1–4]. Hence, peroxisomes balance the generation and degradation of ROS to ensure normal cellular function. The inability to maintain a balance between ROS production and scavenging may induce oxidative stress, which is a major contributor to cellular senescence and pathogenesis of various age-related disorders [1].

Cellular senescence in fibroblasts is characterized by the progressive loss of proliferative capacity with increasing passage number in cells [5]. This is the Hayflick limit in vitro. In early passage, a balance in ROS-generating enzyme (oxidase) and antioxidant exist because ROS generate H_2_O_2_ and the antioxidant catalase decompose those toxic metabolite to water and oxygen. However, as the passage number increases, the defense mechanism of the antioxidant catalase diminishes to counteract the toxic metabolite produced by oxidase and, hence, cell undergo senescence [6]. Similarly, in vivo, mice undergo oxidative damage during aging, diminishing the antioxidant enzyme, particularly catalase [7, 8].

Lysosomes are long-living primary degradative organelle that are responsible for the breakdown of proteins, lipids, and polysaccharides for recycling through the autophagic process [9]. During the autophagic process, the double-membrane autophagosome fuses with lysosome to form autolysosome; hence, the disposable materials are sequestered in the cytoplasm and undergo degradation through lysosomal hydrolases [10]. Lysosomes contain over 50 hydrolases that are activated at low pH (4.5–5.5) and are maintained by the proton-pumping v-ATPase, chloride channels, and ion transporters in the lysosome membrane [11]. Different stress conditions cause lysosomal deterioration, resulting in the translocation of intralysosomal components, such as cathespins, to the cytosol and can induce leaky lysosomes or lysosomal membrane permeabilization (LMP). Aging is a stress condition that alter the physical and chemical properties of lysosomes, increasing their size and in some cases their number also. [12]. Although aging prevents lysosomal acidification and worsens autophagic regulation by the accumulation of long-lived proteins, the mechanism by which the peroxisomal enzyme catalase modulates lysosomes and their effects on aging are not well studied.

The present study investigated the role of catalase and lysosomal function in catalase-knockout (KO) mice. We found that catalase-KO mice exhibited an aging phenotype faster than wild-type (WT) mice. Here, we performed experiments on WT and catalase KO younger (9 weeks) and mature adult (53 weeks) mice [13, 14] and found that KO mice induced an aging phenotype during mature adult; however, we did not observe any phenotypic changes in WT mice at same age (53 weeks). We found that ROS generation in mature adult KO mice might be the main player in inducing a senescence-like phenotype.

## Materials and methods

### Animal treatments

Homozygous KO mice were kindly provided by Dr. Ye-Shih Ho (Institute of Environmental Health Sciences and Department of Biochemistry and Molecular Biology, Wayne State University, USA). The mice were interbred as previously described [15]. Male mice from WT and catalase KO were maintained in accordance with the protocol approved by the Animal Care and Use Committee of Gwangju Institute of Science and Technology, Korea. Mice were fed a standard commercial diet (Research Diet, Inc. NJ, USA) and divided into four groups: (I) WT 9 W (9-weeks-old or young mice), (II) KO 9 W, (III) WT 53 W (53-weeks-old or mature adult mice), and (IV) KO 53 W. Mice were fed a normal chow diet (carbohydrate 65%, protein 20%, and fat 5%), with ad libitum access to water. All experiments were initiated at approximately 9 or 53 weeks of age. Mouse phenotyping studies were performed with controls, and a minimum of two independent experiments were performed with more than five mice in each group.

Mice were sacrificed, and their blood was collected and allowed to clot for 20 min at room temperature (25 °C). Serum was separated by centrifugation at 2000 × g for 20 min in room temperature and stored at –80 °C until further analysis. Liver tissue was removed from each mouse, weighed, frozen in liquid nitrogen, and stored at –80 °C until further use or fixed with formalin. Tissue Sects. (8-µm thick) were used for histochemical analysis.

### Cell culture, reagents, and antibodies

Mouse embryonic fibroblasts (MEFs) from WT and catalase-KO mice were isolated from catalase^+/+^ and catalase^−/−^ mice from E13.5 embryos, as described previously [16]. HepG2 and MEF cell lines were cultured in high-glucose Dulbecco’s modified Eagle medium (DMEM; #11,965,092, Gibco) supplemented with 10% fetal bovine serum (FBS; #16,000,044, Gibco) and 1% penicillin/streptomycin (Invitrogen, Carlsbad, CA, USA) at 37 °C in a 5% CO_2_ incubator. The maximum number of passages for healthy primary MEFs was approximately 5, as cell growth slows beyond passage 5. We worked with MEFs at passages 2 (P2) and 5 (P5) in both WT and KO cells to obtain optimal cellular responses to stimuli [17]. All cell lines were confirmed to be negative for mycoplasma contamination.

Rapamycin (#AY-22989, Selleck Chemicals), NAC (#A7250, Sigma-Aldrich), Lysotracker Red (#L7528, Invitrogen/Thermo Fisher Scientific); MitoSOX Red (#M36008, Invitrogen/Thermo Fisher Scientific), and Leu-Leu methyl ester hydrobromide (LLOMe) (#L7393, Sigma-Aldrich) were added to MEFs and HepG2 cells at the indicated times and concentrations. Indicated antibodies were used as shown in Additional file 1: Table S1.

### Histological analysis

To check the microscopic anatomy of tissues, histological analysis were performed. For this, tissues were fixed in 10% formalin solution, embedded in paraffin, and sectioned. H&E staining was performed as previously described [15].

To analyze the distribution of proteins in cells and tissues based on the use of some fluorescence to visualize the distribution of antibodies, through fluorescence or confocal microscopy, immunofluorescence (IF) was done For IF staining of tissue and cells (both MEFs and HepG2), snap-frozen tissues were sectioned using a cryostat and then defrosted at room temperature for 1 h. Cells and tissue sections were fixed in 4% paraformaldehyde (HT5014; Sigma-Aldrich) at room temperature for 20 min, washed with PBS (Phosphate-Buffered Saline), and incubated with 0.2% Triton X-100 for 5 min. The sections were blocked with 5% goat serum and 0.1% Triton X-100, whereas in vitro, cells were blocked with 3% bovine serum albumin (BSA) for 1 h at room temperature. After blocking, the cells were incubated with the target primary antibody (1:200) with their respective blocking solution overnight at 4 °C. The next day, tissues and cells were washed with PBS and stained with Alexa Fluor-conjugated secondary antibody (1:3000) in blocking solution at room temperature in the dark for 1 h. After that, both tissue and cells were incubated with 10 μM DAPI (4′,6-diamidino-2-phenylindole) in PBS at room temperature for 10 min and mounted with mounting media. Images were acquired and analyzed using an Olympus FluoView 1000 confocal laser scanning system. Slides were scored in a blinded manner. The antibodies used and their sources are listed in Additional file 1: Table S1.

### Oil red O (ORO) staining

To detect the lipid components, ORO staining was done. ORO staining with frozen liver sample was performed as described previously [18]. Briefly, liver tissues were fixed with paraformaldehyde for 20 min and washed with distilled water. ORO solution (01,391; Sigma-Aldrich) was added to the samples for 1 h at room temperature. Sections were washed with distilled water and counterstained with hematoxylin for 1 min. Images were acquired and analyzed using a light microscope. Slides were scored in a blinded manner.

### Measurement of hepatic triacylglycerol (TG)

To detect hepatic TG in experimental mice, TG level was measured. To measure hepatic TG, 50 mg of liver tissue was homogenized, and TG was measured using a triglyceride colorimetric assay kit (#10,010,303, Cayman Chemical) according to the manufacturer’s protocol.

### SA-β-gal staining

To detect senescent or aging cells, SA-β-Gal staining was done. For SA-β-Gal staining in vitro (MEFs and HepG2), cells were washed three times with PBS and stained with senescence β-galactosidase according to the manufacturer’s protocol (#9860, Cell Signaling Technology). For in vivo experiments, both WT and catalase-KO mice at 9 W and 53 W were perfused through the heart initially with PBS, followed by 4% paraformaldehyde. Liver tissues were collected, frozen sectioned, and stored at − 80 °C for future use. Senescence β-galactosidase staining was performed according to the manufacturer’s protocol (#9860, Cell Signaling Technology). Images were acquired and analyzed using a light microscope. Slides were scored in a blinded manner. SA-β-Gal staining intensity was determined using the ImageJ software, and the mean SA-β-Gal intensity in the region of interest was measured based on the cell shape. SA-β-Gal positivity was determined as fold-change relative to the control.

### Lysotracker red and MitoSOX staining

To detect acidic probes of lysosomes and mitochondrial ROS, in the form of fluorescence, lysotracker and mitosox staining was done respectively. Those staining were performed in paraformaldehyde fixed cells, according to the manufacturer’s instructions. Images were acquired using a fluorescence microscope, with the same fixed exposure time for all samples in each experiment.

### Western blot analysis

To determine the expression levels of target proteins using gel electrophoresis western blotting from whole cell analysis,tissue samples and MEFs were performed as described previously [15]. The antibodies used and their sources are listed in Tab s1.

### Cell fractionation

To separate and isolate subcellular components from one aonother, cell fractionation was done. It was performed to check cathespin protein (in either the membrane or cytosol) in both cell and liver tissues, as described previously [19]. In brief, treated cells and fresh liver tissues were washed with ice-cold PBS and homogenized with homogenization buffer [10 mM HEPES, 0.25 M sucrose, 10 mM Na_2_EDTA, adjusted to pH 7.0 with NaOH, and 1 × protease inhibitor cocktail (GenDEPOT, P3100-001; P3200-001)]. After homogenization, both tissues and cells were centrifuged at 1000 × g for 10 min, and post-nuclear supernatants were further centrifuged at 100,000 × g for 1 h to generate supernatant and pellet fractions. All the procedures were performed at 4 °C. After measuring the protein concentration, the supernatant and pellet extracts were boiled at 97 °C for 10 min and subjected to western blot analysis.

### Determination of ROS production

To check the superoxide radical (O• − 2) or H_2_O_2_ in tissues, Reactive oxygen species (ROS) was measured. ROS production from tissue lysates and cells was performed as previously described [15].

### ACOX1 activity

To check the level of ACOX1 (acyl-CoA oxidase 1), a major producer of ROS in the peroxisome, ACOX1 protein level by ELISA assay kit was measured. Liver tissues were homogenized in ice-cold PBS. The homogenates were centrifuged and supernatants were collected, and the levels of acyl-CoA oxidase 1 (ACOX1) were measured using ELISA assay kits (My Biosource).

### Cathespin D activity

To check the intralysosomal components, Cathespin D activity was measured. Treated cells and fresh liver tissues were washed with ice-cold PBS and homogenized with lysis buffer provided by a commercially available kit (#ab65302, Abcam). The activity of cathespin D was performed as described in kit.

### Statistical analysis

Data are presented as means ± standard deviation (SD). One-way analysis of variance was used to compare the means of two or more groups. Statistical significance was set at *P* < 0.05.

## Results

### Catalase-deficient mice induce aging phenotype faster than WT mice

**Fig. 1:**
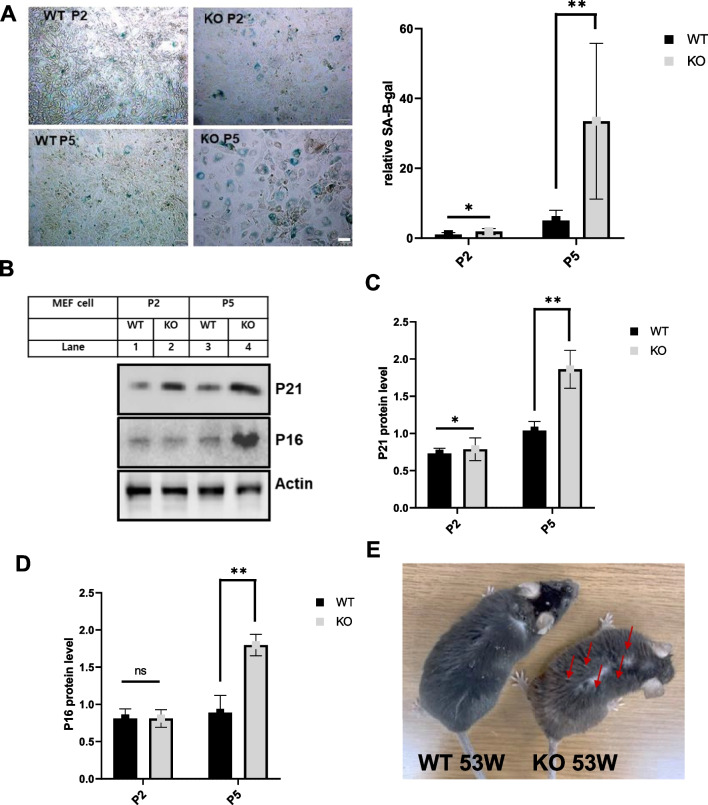

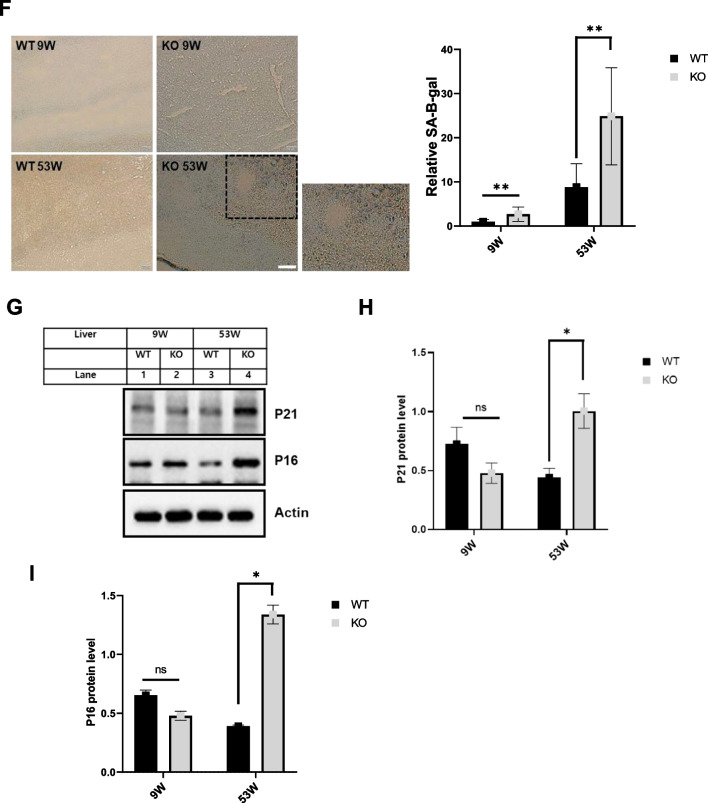
Catalase-deficient mice induce aging phenotype faster than WT mice. **A** Morphology and senescence-associated β-galactosidase staining of WT and catalase KO MEFs at passage 2 (P2) and passage 5 (P5) levels. Intensities of senescent cells at each passage, P2 and P5, in WT and KO MEFs. Positive intensities of β-galactosidase staining were measured compared to control using the ImageJ software. Scale bar represents 100 μm. **B** Proteins were extracted from MEFs at P2 and P5 level. Immunoblot analysis was performed using whole-cell lysates with indicated senescence-associated antibody. **C**, **D** quantified protein level of P21 and P16 normalized with actin. **E** Morphology of dorsal back hair of WT and KO mice at 53 W. **F** Senescence-associated β-galactosidase staining was performed in the liver section of 9 weeks (9 W) and 53 weeks (53 W) WT and catalase-KO mice. Intensities of senescent cells. Positive intensities of β-galactosidase staining were measured compared to control using the ImageJ software. Scale bar represents 100 μm. **G** Immunoblot analysis was performed from the liver sample from WT and catalase-KO mice at the age of 9 W and 53 W with indicated antibody. **H**, **I** quantified protein level of P21 and P16 normalized with actin. Bar graph represents mean ± SD (n = 3 experiments). *P < 0.05; **P < 0.0001; ns: non-significant

It has been well documented that increased ROS and diminished antioxidant capacity induce cellular senescence, and catalase enzymes have been used to alleviate senescence through its antioxidant defense mechanism [6, 8, 20]. We investigated whether catalase is used to alleviate ROS and diminish aging in catalase-deficient mice. For this, WT and catalase-KO mouse embryonic fibroblasts cells (MEFs) were isolated and cultured. Catalase deficiency was confirmed in KO MEFs by immunoblot analysis with anti-catalase (Additional file 1: Fig. S1a). KO MEFs displayed flattened and enlarged senescence phenotypic morphology at early passage (P2) and showed increased senescence phenotypic morphology with increasing passage (P4) (Additional file 1: Fig. S1b). To determine whether the flattened and enlarged MEFs were senescent, β-galactosidase staining was performed. As expected, KO MEFs showed positive staining for senescence-associated β-galactosidase, which significantly increased in P5 but not in WT cells (Fig. 1A and Additional file 1: Fig. S1c). To illustrate the senescence-induced phenotype, immunoblot analysis was performed for WT and KO MEFs from P2 and P5. Notably, the expression levels of senescence-related proteins p21 and p16 increased in KO P5 MEFs (Fig. 1B–D). Furthermore, to confirm the aging phenotype, catalase-KO mice with WT littermates at the age of 9 weeks (9 W) and 53 weeks (53 W) were subjected to the experiment. Partial hair in the dorsal back skin of 53 W KO mice turn gray whereas it looks normal in WT mice (Fig. 1E, indicated by red arrow). β-galactosidase staining was performed in the liver of mice, which showed positive staining in KO mice livers at 53 W (Fig. 1F). Immunoblot analysis of liver homogenates from mice showed the induction of senescence-related proteins in KO mice at 53 W (Fig. 1G–I). Together, these data suggest that catalase deficiency induces an aging phenotype faster than WT mice.

### Catalase-deficient mice induce ROS through mitochondria and peroxisome

**Fig. 2:**
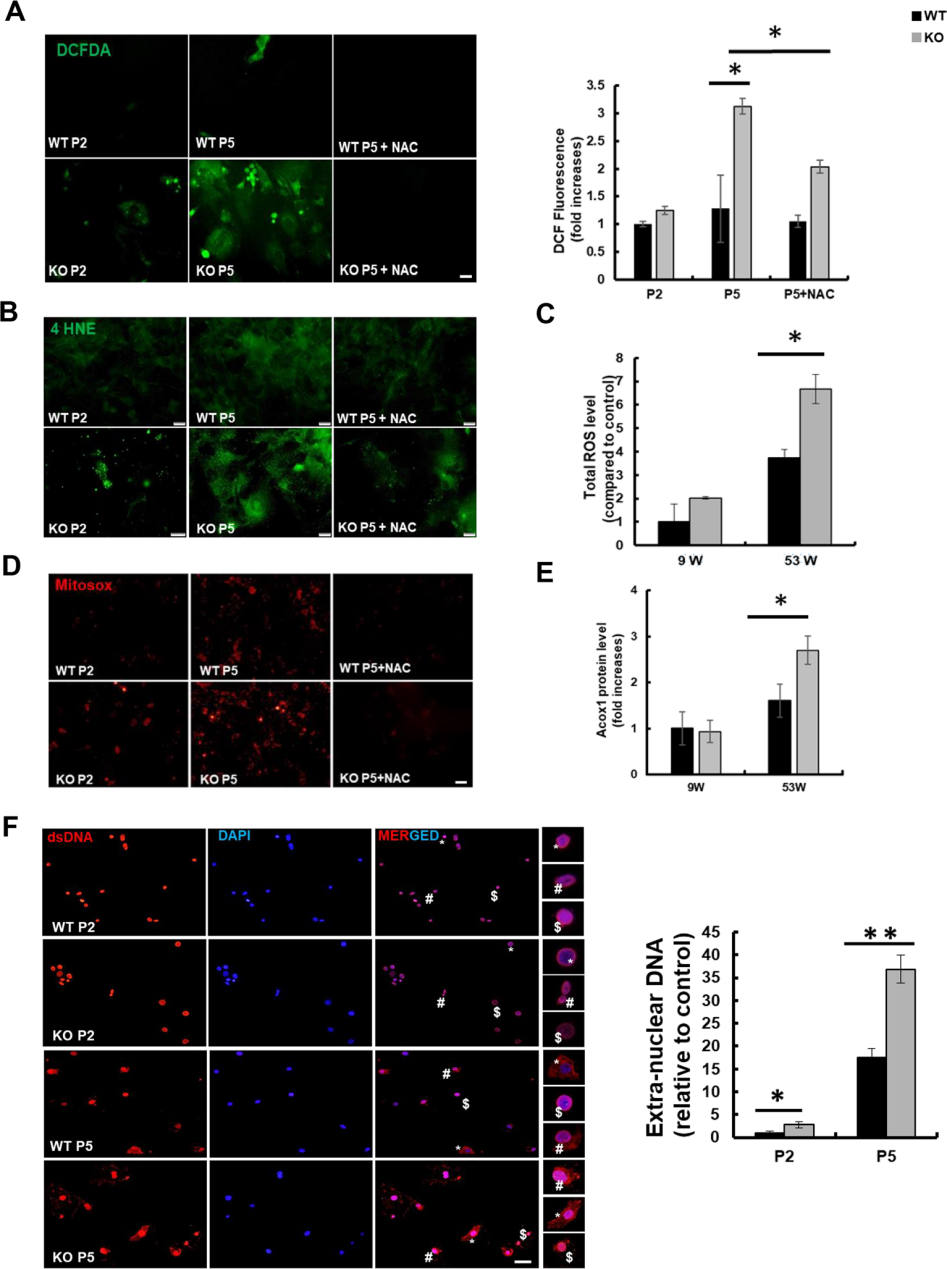

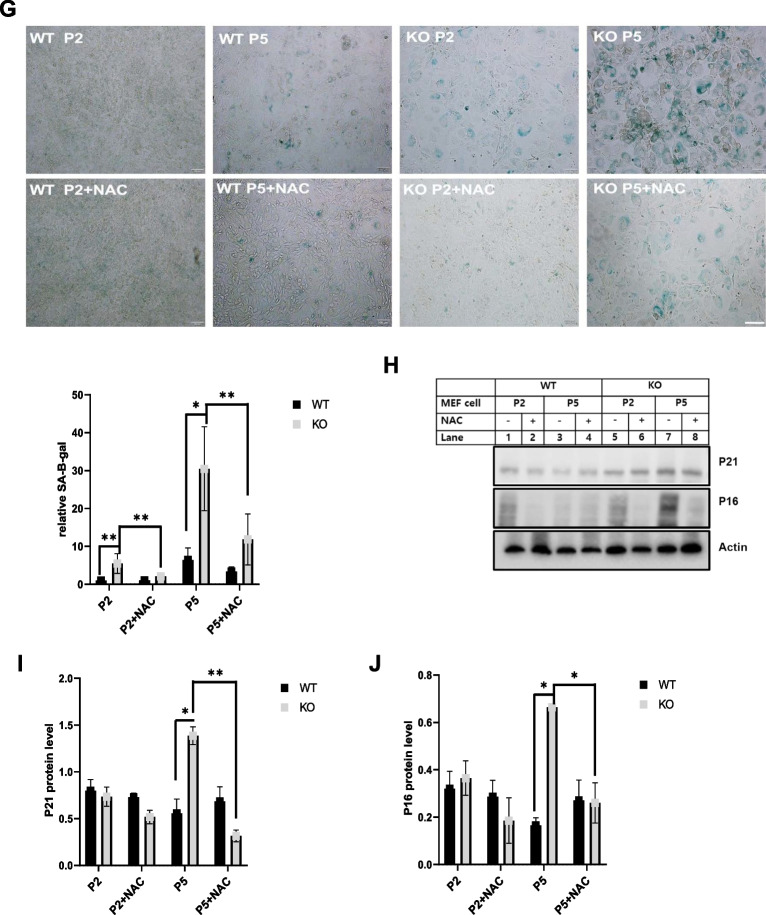
Aging is induced in catalase-deficient mice through ROS generation. **A** Representative fluorescence images of DCFH-DA staining of WT and catalase-KO MEFs at P2 and P5 levels. Quantification of cells showing green fluorescence (corresponding to DCFH-DA) and fluorescence intensity of MEFs. *P < 0.05 WT P5 versus KO P5; KO P5 versus KO P5 + NAC. **B** Representative fluorescence images of MEFs fixed and immunostained with anti-4HNE (green). Scale bar represents 20 μm. **C** Total ROS was measured in liver lysates of WT and catalase-KO mice at 9 W and 53 W. Values represent mean ± SD (n = 3, 4). *P < 0.05 WT 53 W versus KO 53 W. **D** Representative (red) fluorescence image of MitoSOX staining in MEFs, as in **A**. **E** ACOX1 levels were measured using the ACOX1 ELISA kit from the liver lysates of mice as in C. **F** IF staining of anti-dsDNA (red), DAPI (blue) in MEFs indicated as in A. Insets(*,#, $) enlarged cells; scale bar, 20 μm. Intensities of dsDNA positive cells at each passage, P2 and P5, in WT and KO MEFs.**G** MEFs at P2 and P5 were treated with 5 mM NAC overnight, and senescence-associated β-galactosidase staining was analyzed. The percentage of senescent cells was analyzed in WT and KO MEFs treated with NAC. Positive intensities of β-galactosidase staining were measured using the ImageJ software. Bar graph represents mean ± SD (n = 3 experiments). Scale bar represents 100 μm. *P < 0.05, WT P2 versus KO P2; KO P2 versus KO P2 + NAC; WT P5 versus KO P5. KO P5 versus KO P5 + NAC. **H** Proteins were extracted from MEFs as in G. Immunoblot analysis was performed using whole-cell lysates with indicated senescence-associated antibodies. **I**, **J** quantified protein level of P21 and P16 normalized with actin. Bar graph represents mean ± SD (n = 3 experiments). *P < 0.05; **P < 0.0001

As reported earlier, ROS generation is the main cause of aging due to decreased cellular antioxidant capacity [6, 8, 20]. We hypothesized that ROS generation might be increased in catalase-KO mice. As expected, the fluorescence intensity measured by 2′7′-dichlorofluorescein diacetate (DCFH-DA) staining showed an increase in catalase-KO MEFs at P5 (Fig. 2A). The fluorescent signal of DCFH-DA staining, representing ROS, was quantified and showed that ROS generation was significantly higher in catalase-KO cells at P5 (Fig. 2A). Likewise, we examined the expression of another ROS marker, 4 hydroxynonenal (4-HNE), in MEFs by immunofluorescence staining (IF). Consistent with DCFH-DA staining, the fluorescence intensity of 4-HNE in KO MEFs increased at P5 (Fig. 2B). In addition, to confirm ROS generation in vivo, intracellular ROS levels were measured in liver lysates of mice. Total ROS levels increased significantly in KO mice at 53 W (Fig. 2C). The endogenous source of ROS contains different cellular locations, with mitochondria and peroxisomes being the major sites [2, 21, 22]. The principal source of ROS produced by mitochondria is the superoxide anion, a byproduct of the electron transport chain responsible for oxidative damage by aerobic energy metabolism [23, 24]. To detect mitochondrial ROS, MitoSOX red, a mitochondrial superoxide indicator, was used in WT and KO MEFs at the P2 and P5 levels (Fig. 2D). Catalase-KO cells at P5 showed increased levels of the red fluorescence signal. In addition, in vivo, the level of ACOX1 (acyl-CoA oxidase 1), a major producer of ROS in the peroxisome and the first and rate-limiting enzyme in fatty acid β-oxidation, increased significantly in KO mice at 53 W (Fig. 2E). To confirm the induction of ROS in catalase-KO mice, MEFs were treated with the antioxidant N-acetyl-L-cysteine (NAC). Treatment with NAC, however, inhibited the level of ROS generation in KO MEFs at P5 (Fig. 2A, B, and D). Taken together, these data suggest that catalase-KO mice induce ROS production through both mitochondria and peroxisomes. Generation of ROS is susceptible to lipid oxidation and protein carbonylation that lead to DNA degradation, indicating nucleic acid oxidative damage [25]. DNA released from nucleus to cytosol after oxidative damage contributes to senescence [26]. Based on this, Using double-stranded DNA (dsDNA) antibodies to detect DNA by immunofluorescence (IF), we found that almost 30% of KO MEFs at P5 exhibited extra nuclear DNA in contrast to 15 and 5% in WT P5,P2 and KO P5 cells (Fig. 2F).Taken together, it shows that extranuclear DNA in KO MEFs regulate aging through ROS.

We hypothesized that ROS generation in catalase-KO mice may induce cellular senescence, as previously described [6, 8, 20]. Hence, β-galactosidase staining was again performed in WT and catalase-KO MEFs and co-treated with NAC (Fig. 2G). As expected, positive staining of senescence-associated β-galactosidase in KO MEFs was significantly diminished by treatment with NAC. Moreover, NAC treatment also decreased senescence-related protein in KO P5 MEFs (Fig. 2H–J). Together, these data suggest that catalase deficiency induces an aging phenotype through ROS generation.

### Catalase-deficient mature adult mice induce leaky lysosome

**Fig. 3:**
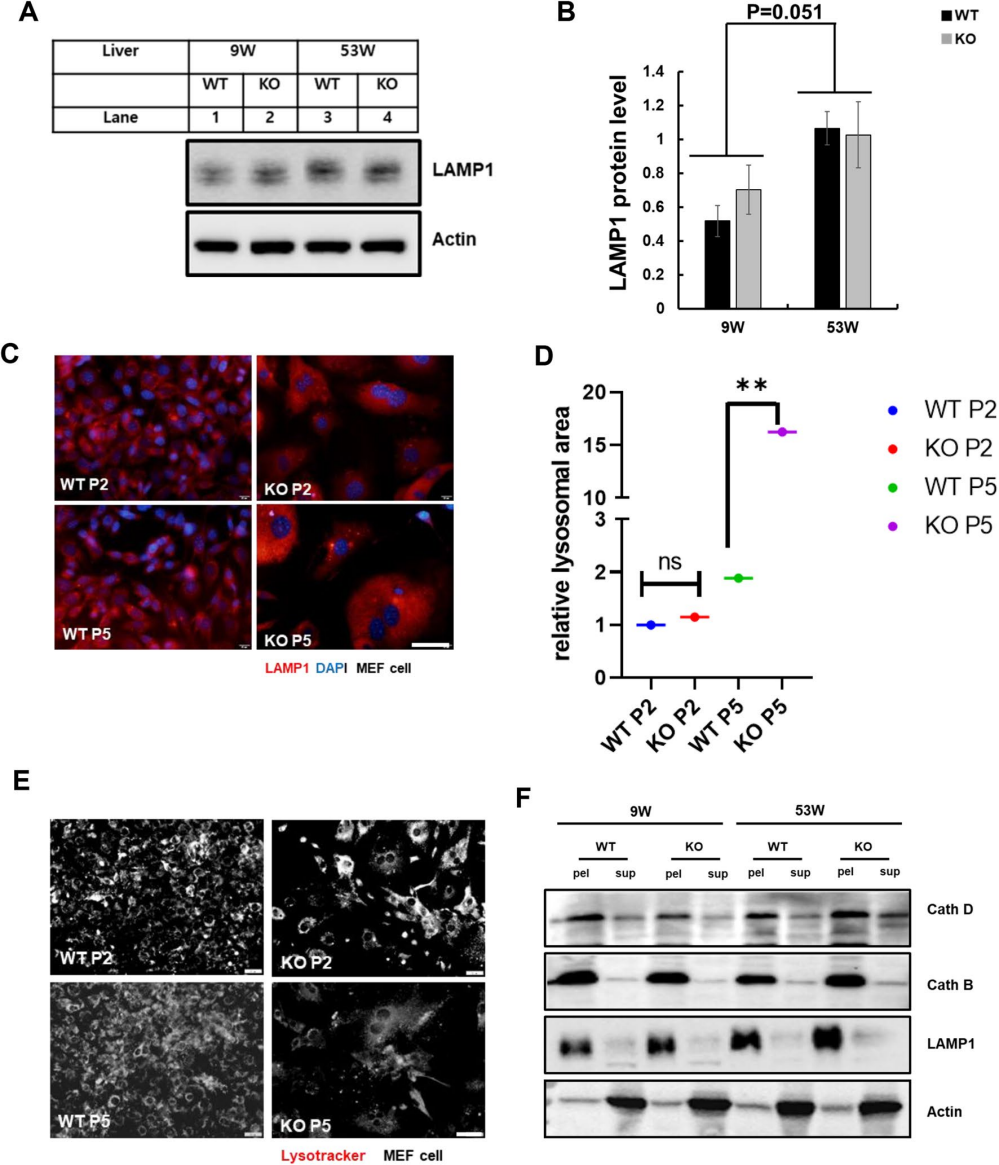

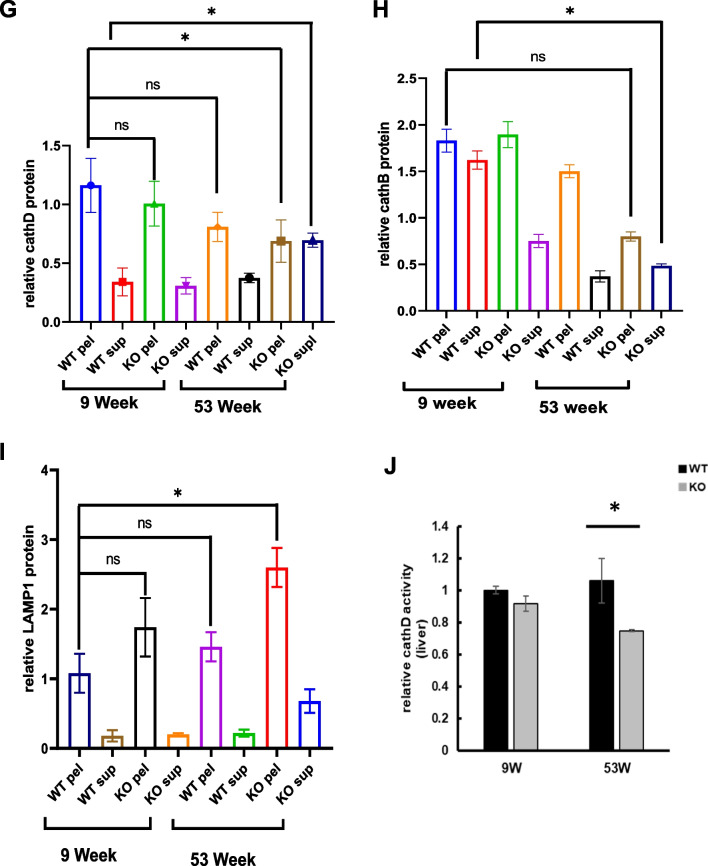
Catalase-deficient mature adult mice induce leaky lysosomes. **A** Protein was extracted from liver lysates from WT and catalase-KO mice at 9 W and 53 W.Immunoblot analysis was performed using whole-cell lysates with LAMP1 antibody. **B** Quantified protein level of LAMP1 normalized with actin. **C** Representative fluorescence images of MEFs fixed and immunostained with anti-LAMP1 (red) and DAPI (blue). Scale bar represents 20 μm. **D** Intensities of lysosomal area showing red fluorescence (corresponding to control) and fluorescence intensity of MEFs. **E** Representative fluorescence images of lysotracker for WT and catalase-KO MEFs at P2 and P5 levels. Scale bar represents 20 μm. **F** Liver lysates from WT and catalase-KO mice at 9 W and 53 W were fractioned as described in the Materials and Methods. Equivalent volumes of each fraction were subjected to immunoblotting using the indicated antibodies. **G**–**I** Quantified protein level of cathD, cathB and LAMP1 on pellet and supernatant fractionations, normalized with actin. **J** Relative cath D levels were measured from liver lysates of mice, as indicated in A. Bar graph represents mean ± SD (n = 3 experiments). *P < 0.05; **P < 0.0001; ns: non-significant

Lysosomes are the main catabolic organelle that play an essential role in cellular process, including response to nutrient availability, stress resistance, plasma membrane repair development, and cellular differentiation [27]. In line with catabolic organelles, lysosomal activity is strongly influenced by aging by altering the physical and chemical properties of these organelles and rendering them more sensitive to stress [12]. Considering this notion, immunoblot analysis of mice liver homogenates was performed to check the lysosomal marker protein LAMP1 (Fig. 3A). Lysosomal protein levels was significantly increased in both the mice liver at the age of 53 W (Fig. 3A, B). Aging has also been reported to increase lysosomal volume [28]. Hence, to check the volume, the morphology of lysosomes was analyzed using immunofluorescence (IF). WT and KO MEFs were immunostained with the lysosomal marker LAMP1 (Fig. 3C). Catalase-deficient MEFs at the P5 level showed an enlarged cellular size, which significantly increased the red fluorescence signal toward the cytoplasm of the cell. To confirm the lysosomal area, the fluorescent signal of LAMP1 was quantified and showed that lysosomal area was significantly higher in catalase-KO MEFs at P5 (Fig. 3D). Again we questioned, did increased in the lysosomal area affect the acidity of lysosme? Hence to confirm this, MEFs were immunostained with lysotracker for labeling and tracking of the acidotropic probe for lysosomes (Fig. 3E). The fluorescence intensity of acidic vesicle specifically accumulating in lysosomes decreased, in KO MEFs at the P5 level (Fig. 3E). Lysosomal activity is highly influenced by hydrolytic enzymes residing in the lumen of the lysosomal membrane, which is highly acidic [19]. As the acidotropic probe for lysosomes decreased in KO MEFs, we assumed that the resident hydrolytic enzymes in the lysosomal lumen were leaked. Hence, leaky lysosomal content may make the hydrolytic enzyme alkaline, causing lysosomes to fuse in the cytosol, which may increase lysosomal size. To check the lysosomal content, we analyzed the protein levels of cathepsin D (cathD) and cathepsin B (cathB), two major lysosomal hydrolases that can serve as molecular reporters for lysosomal functions, in WT and KO liver homogenates (Fig. 3F). Both lysosomal hydrolases were accumulated in the supernatant fraction in KO mice at 53 W, whereas both cath D and B were normal in the pellet fraction in the other groups (Fig. 3F–H). Again LAMP1 protein was significantly increased in the pellet of KO mice liver at the age of 53 W (Fig. 3F, I). Furthermore, cath D activity was measured in the liver lysates of mice (Fig. 3J). cath D activity significantly decreased in KO mice at 53 W. Together this data confirmed that catalase KO mice at 53 W induce leaky lysosome causing accumulation of lysosomal hydrolases in the cytoplasm that make hydrolytic enzyme alkaline and increase lysosomal volume towards the cytoplasm.

### Leaky lysosome persuades alteration of autophagy through ROS in catalase-deficient mature adult mice

**Fig. 4:**
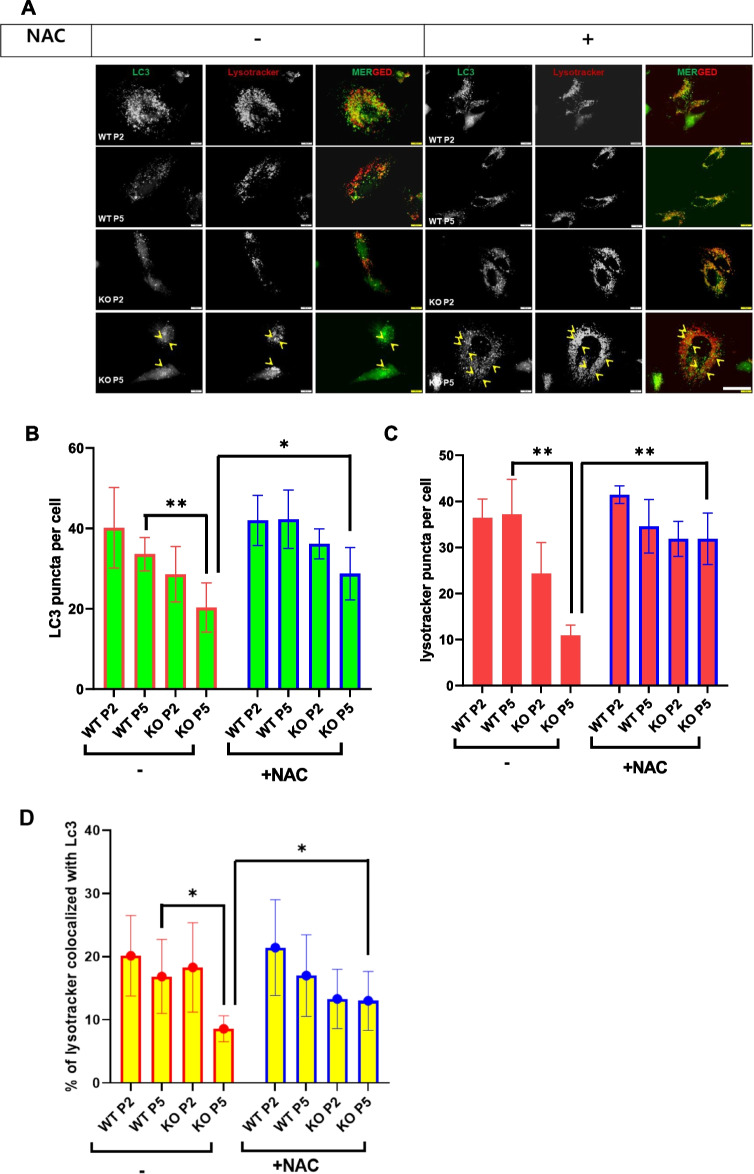

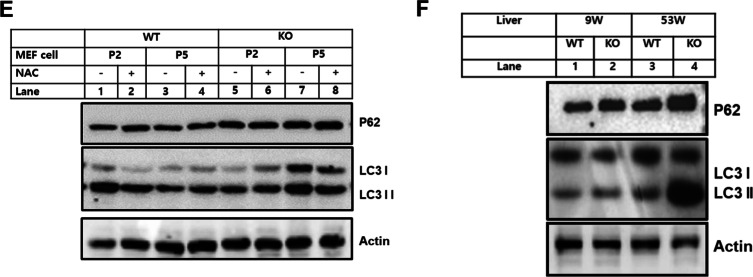
Catalase-deficient mature adult mice induce alteration of autophagy through ROS. **A** MEFs from WT and catalase-KO mice at P2 and P5 levels were treated with 5 mM NAC, fixed, and immunostained with anti-LC3 (green),Lysotracker (red), yellow showed LC3 puncta co-localized to lysosme (arrow head). Scale bar represents 10 μm. Quantification of **B** LC3, **C** lysotracker and **D** co-localized cell analyzed from each experimental group as in **A**. The bar graph represents the mean ± SD (n = 3 experiments). *P < 0.05; **P < 0.0001. **E** Proteins were extracted from MEFs as in A. Immunoblot analysis was performed using whole-cell lysates with the indicated antibodies. **F** Protein was extracted from liver lysates from WT and catalase-KO mice at 9 W and 53 W. Immunoblot analysis was performed using whole-cell lysates with the indicated antibodies

Damaged lysosomes are selectively sequestered by autophagy [19]. Hence, we hypothesized that damaged lysosomes in KO mice may be recruited by autophagy machinery, which are then engulfed by autophagosomes. To confirm the autophagic process, MEFs were co-immunostained with LC3, an autophagy marker, with lysotracker (Fig. 4A). In contrast to our assumption, the LC3 positive puncta green fluorescence decreased and were not fused with lysosomes in KO MEFs due to decreased lysosomal acidic probe, which showed less fusion of autophagosomes with lysosomes (Fig. 4A–D). Further, immunoblot analysis was performed in WT and KO MEFs at the P2 and P5 levels. Notably, the expression levels of autophagy substrate marker P62 and autophagosome by LC3II were significantly increased in KO MEFs at P5 (Fig. 4E). To confirm autophagic dysregulation in vivo, liver tissues from mice were immunoblotted with an autophagy marker (Fig. 4F). Consistent with MEFs, liver tissues from KO mice at 53 W showed dysregulation of basal autophagy in comparison to WT and KO 9 W livers (Fig. 4F). It’s known that ROS mediate leaky lysosomes [29]. Hence, we hypothesize that ROS might be the main player to dysregulate autophagy through leaky lysosome. Hence, to confirm this, WT and catalase-KO MEFs were treated with or without NAC at both passages (P2 and P5). Treatment with NAC slightly recovered the acidic probe of lysosomes, which fused with autophagosomes in KO MEFs at the P5 level (Fig. 4A–E). Hence, these data suggest that leaky lysosomes alter autophagy through ROS in catalase-deficient mature adult mice.

### Leaky lysosomes induce lipofuscin accumulation through ROS in catalase-deficient mature adult mice

**Fig. 5:**
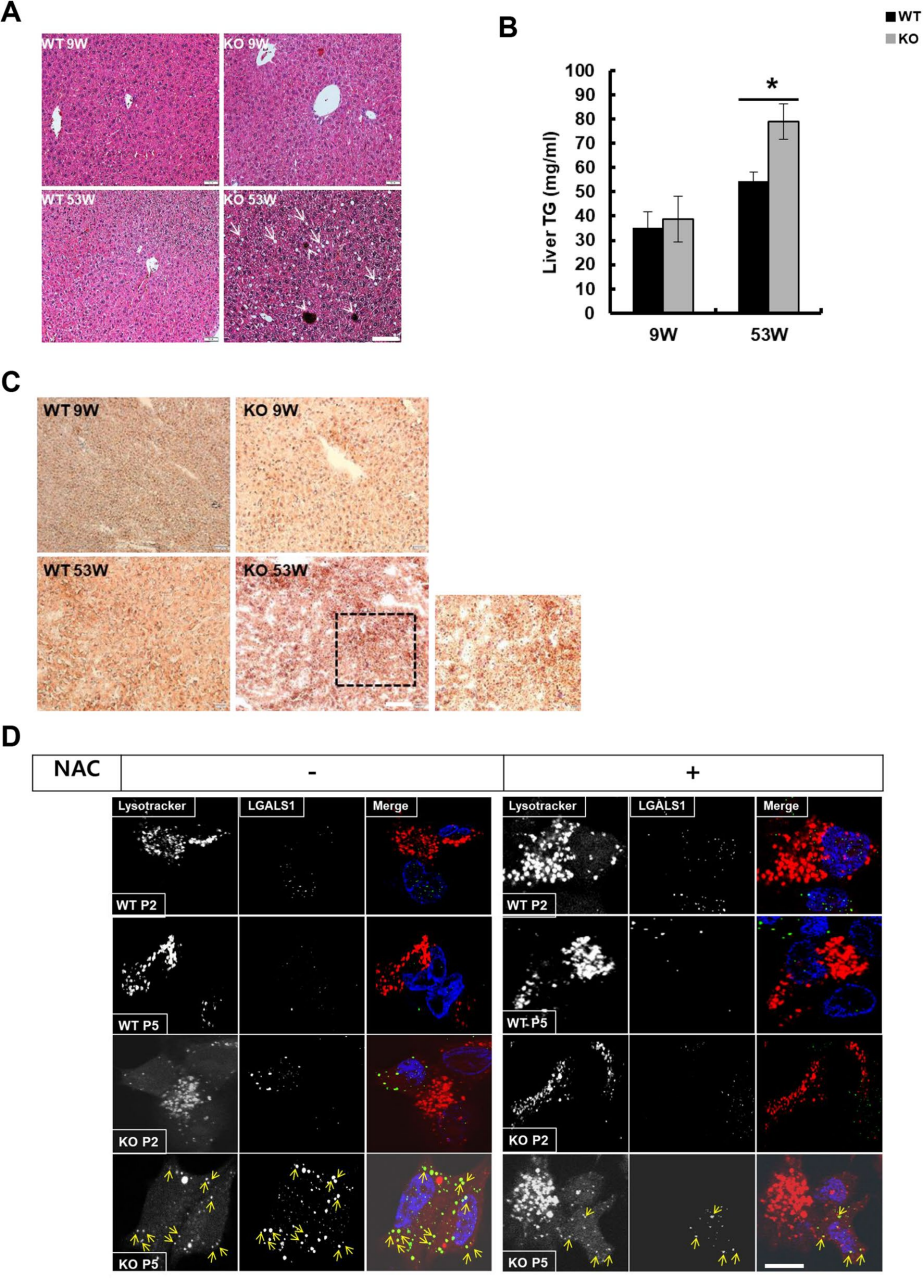
Catalase-deficient mature adult mice induce lipofuscin accumulation through ROS. **A** Representative hematoxylin and eosin (H&E) staining of livers from WT and catalase-KO mice at the age of 9 W and 53 W, respectively. The livers of 53 W KO mice showed microvesicular steatosis (i.e., accumulation of small fat droplets) in the cytosol of hepatocytes (arrows) with a golden-brown pigment (arrowhead) known as lipofuscin. Scale bar represents 50 μm. **B** Liver samples from mice as mentioned in** A** were homogenized, and TG levels were analyzed. Bar graph represents mean ± SD (n = 3 experiments). *P < 0.05, WT 53 W versus KO 53 W. **C** Cryosectioned liver tissues from mice were stained with ORO. Scale bar represents 50 μm. **D** Representative fluorescence images of MEFs at P2 and P5 treated with 5 mM NAC, fixed, and immunostained with Lysotracker (red), LGALS1 (green), and DAPI (blue). Scale bar represents 5 μm

Although leaky lysosomes are induced in catalase-deficient MEFs, the damaged lysosomes were not degraded through the autophagic process. Instead, basal autophagy was dysregulated in KO MEFs. To find the mechanistic evidence, we examined the morphology of the liver by H&E staining in mice. The liver morphology of 53 W KO mice showed microvesicular steatosis (i.e., accumulation of small fat droplets) in the cytosol of hepatocytes (arrows), with brown pigment (arrowhead), whereas the livers of WT and KO 9 W mice showed normal lobular architecture with hepatocytes arranged in hepatic cords (Fig. 5A). To confirm the accumulation of fat droplets in hepatocytes, triacylglycerol (TG) was measured in the liver lysates of mice. Consistent with H&E staining, liver lysates of 53 W KO mice showed a significant increase in liver TG compared to other group (Fig. 5B). Furthermore, oil red O staining (ORO) also showed the induction of lipid droplets in the hepatocytes of 53 W liver sections (Fig. 5C). During aging, the volume and structure of hepatocyte organelles change [30]. Although we observed a significant increase in body weight of 53 W old KO mice, but there were no significant changes in liver weight (Additional file 1: Fig. S2a, b). We hypothesized that the accumulation of small lipid droplets in the cytoplasm of hepatocytes in KO 53 W old mice may be an undigested lipid, lipofuscin, which showed brown pigmentation on hepatocytes (Fig. 5A, white arrow head). Lipofuscin is a highly oxidized insoluble protein that fails to degrade damaged and denatured proteins [31]. Moreover, it is a chemically and morphologically polymorphous waste material that accumulates at the primary site of the lysosome and disturbs lysosomal degradation and causes lysosome leakage [32, 33]. To examine the accumulation of lipofuscin or leaky lysosomes, MEFs were co-immunostained with LGALS1 (galectin-1), a leaky lysosome marker with lysotracker (Fig. 5D). Catalase-KO MEFs at P5 level showed a significant increase in LGALS1 puncta that were loaded on the lysosomes (Fig. 5D, arrow); although acidic vesicles in lysosomes by lysotracker were less, almost all LGALS1 puncta were localized to lysosomes. In contrast, LGALS1 puncta were less or not observed at all in the WT at P2, P5, and KO P2 levels. It is known that enhanced ROS results in the leakage and accumulation of lipofuscin in lysosomes [29, 33]. Hence, to confirm this, WT and catalase-KO MEFs were treated with or without NAC at both passages (P2 and P5). Treatment with NAC slightly rescued the acidic vesicles of lysosomes and decreased the localization of LGALS1 puncta to the lysosomes in KO MEFs at P5 level (Fig. 5D). Together, these data suggest that leaky lysosomes induce lipofuscin accumulation through ROS in catalase-KO mature adult mice.

### Leaky lysosome affects lysosomal pH that activates mTORC1 and leads to cellular senescence

**Fig. 6:**
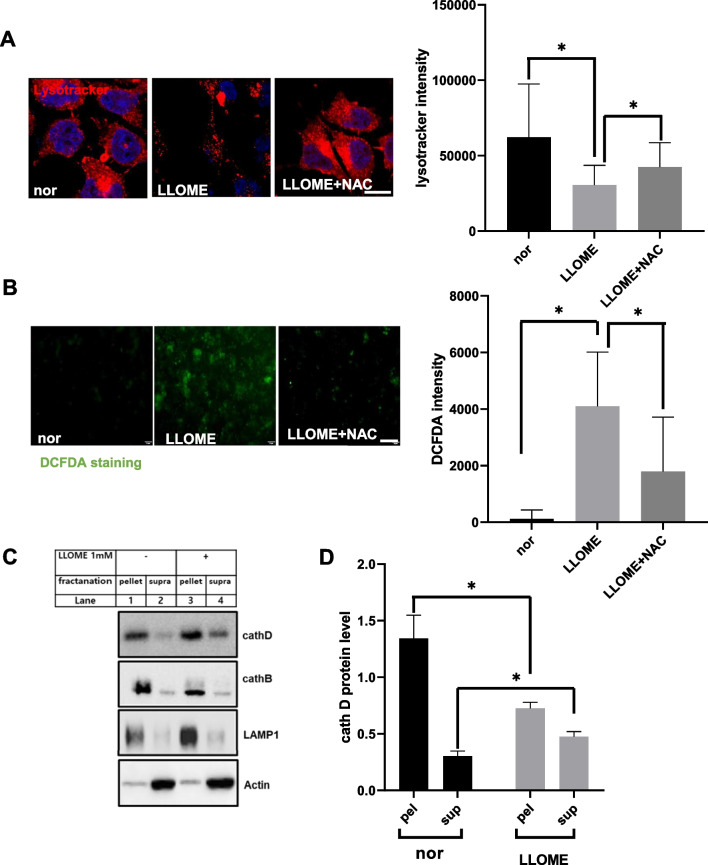

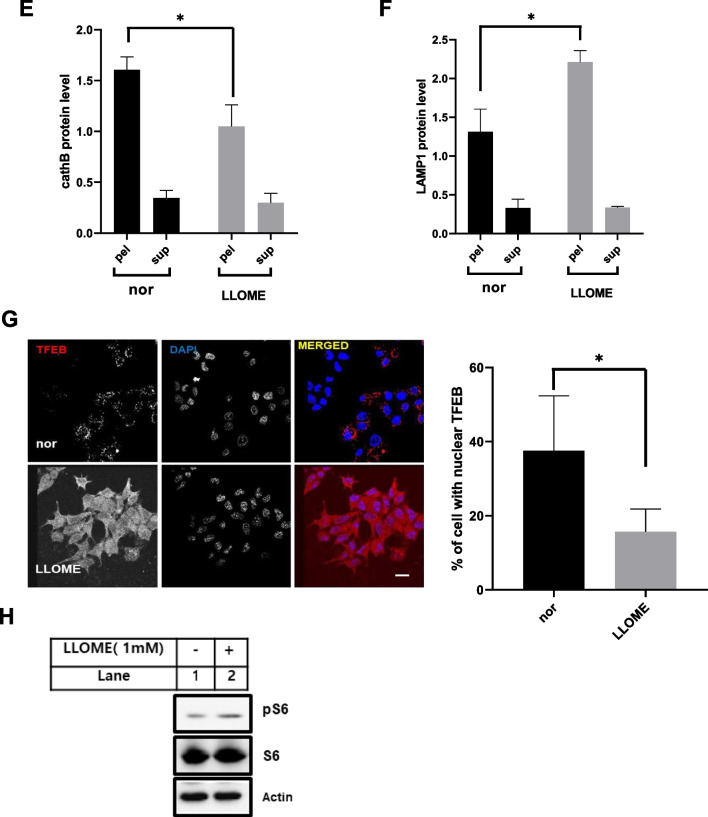
Catalase-deficient mature adult mice induce mTORC1 activation. **A** HepG2 cells were treated with 1 mM LLOMe for 24 h and co-treated with 5 mM NAC overnight. Representative fluorescence images of HepG2 cells immunostained with Lysotracker (red) and DAPI (blue). Scale bar represents 5 μm. **B** Representative fluorescence images of DCFH-DA staining of HepG2 cells treated as in a. Scale bar represents 20 μm. **C** Lysates of HepG2 cells treated as in A were fractioned as explained in the Materials and Methods section. Equivalent volumes of each fraction were subjected to immunoblotting using the indicated antibodies. **D**–**F** Quantified protein level of cathD, cathB and LAMP1 normalized with actin. **G** Representative fluorescence image of HepG2 cells treated as in A, immunostained with anti-TFEB (red) and DAPI (blue). Scale bar represents 20 μm. Quantified bar represents percentage of nuclear localization of TFEB on treated cell as indicated. **H** Protein was extracted from HepG2 cells as in a. Immunoblot analysis was performed using whole-cell lysates with indicated antibodies. Bar graph represents mean ± SD (n = 3 experiments). *P < 0.05; **P < 0.0001

Next, we constructed leaky lysosomes using the well-known lysosomal membrane permeabilization (LMP) marker L-leucyl-L-leucine methyl ester (LLOME) in hepatoma cells and questioned whether leaky lysosomes induce ROS and cellular senescence. For this, we treated HepG2 cell with LMP inducer LLOME for 24 h and examined the morphology of lysosomes by immunostaining with lysotracker. As expected, the specific accumulation of acidic vesicles in lysosomes decreased in LLOME-treated cells (Fig. 6A). The disruption of acidic hydrolases in lysosomes or leaky lysosomes is induced through extensive ROS [29]. To confirm this, HepG2 cells were stained with DCFH-DA (Fig. 6B). Fluorescence intensity by DCFH-DA staining increased in LLOME-treated cells. Hence, to confirm the leaky lysosomes induced by ROS accumulation, HepG2 cells were co-treated with antioxidant NAC in LLOME treated cells. As expected, NAC significantly recovered the acidic vesicles of lysosomes and inhibited DCFH-DA fluorescence intensity in HepG2 cells (Fig. 6A–B). LLOME treatment increased the cytosolic release of lysosomal hydrolases [19]. Hence, to check the lysosomal content, the protein levels of cath D and B were immunoblotted in HepG2 cells treated with LLOME (Fig. 6C). As expected, both lysosomal hydrolases (cath D and B) accumulated in the supernatant fraction in LLOME-treated cells, whereas they were normal in the pellet fraction of untreated cells (Fig. 6C–E). Also the protein level of lysosome significantly increased in LLOME treated cell (Fig. 6C, F). Further immunoblot analysis of HepG2 cells showed increased protein expression of the ROS marker 4-HNE and peroxisomal oxidase ACOX1 in LLOME-treated cells (Additional file 1: Fig. S3a). However, we did not observe any changes in mitochondrial enzymes, including COX1, COX4, voltage-dependent anion channel (VDAC), and antioxidant proteins, including SOD1 and SOD2.

The link between decreased lysosomal function and aging has been well studied [12, 34]. Hence, we investigated whether LMP drug LLOME induces cellular senescence in cells. β-galactosidase staining was performed in HepG2 cells. As expected, cells treated with LLOME showed positive staining for senescence-associated β-galactosidase (Additional file 1: Fig. S3b). However, treatment with NAC inhibited the positive staining of senescence-associated β-galactosidase. Further immunoblot analysis was performed in HepG2 cells showing increased expression of aging-related proteins, including p16 and p21, in LLOME-treated cells (Additional file 1: Fig. S3c). During lysosomal damage, transcription factor EB (TFEB), a major regulator of autophagy and lysosomal biogenesis, has been shown to rapidly translocate to the nucleus and activate the transcription of its target gene for the activation of lysosomes [35]. Hence, immunostaining was performed for translocation of TFEB to HepG2 cells. As expected, LLOME-treated cells showed translocation of TFEB to the nucleus in LLOME-treated cells (Fig. 6d). Hence, lysosomal rupture induces the biogenesis of lysosomes through autophagy (Additional file 1: Fig. S3d), as previously described [19, 35]. Meanwhile, immunoblot analysis also showed that treatment with LLOME increased the expression of phosphorylated S6 (pS6), a marker of downregulation of mTORC1 (Fig. 6H). Further immunostaining with anti-mTORC1was performed in LLOME-treated cells, which showed increased expression of mTORC1 protein (Additional file 1: Fig. S3e). Together, these data show that leaky lysosomes affect lysosomes and induce cellular senescence probably through mTORC1 activation.

### Rapamycin attenuated cellular senescence induced by catalase-deficient cells

**Fig. 7:**
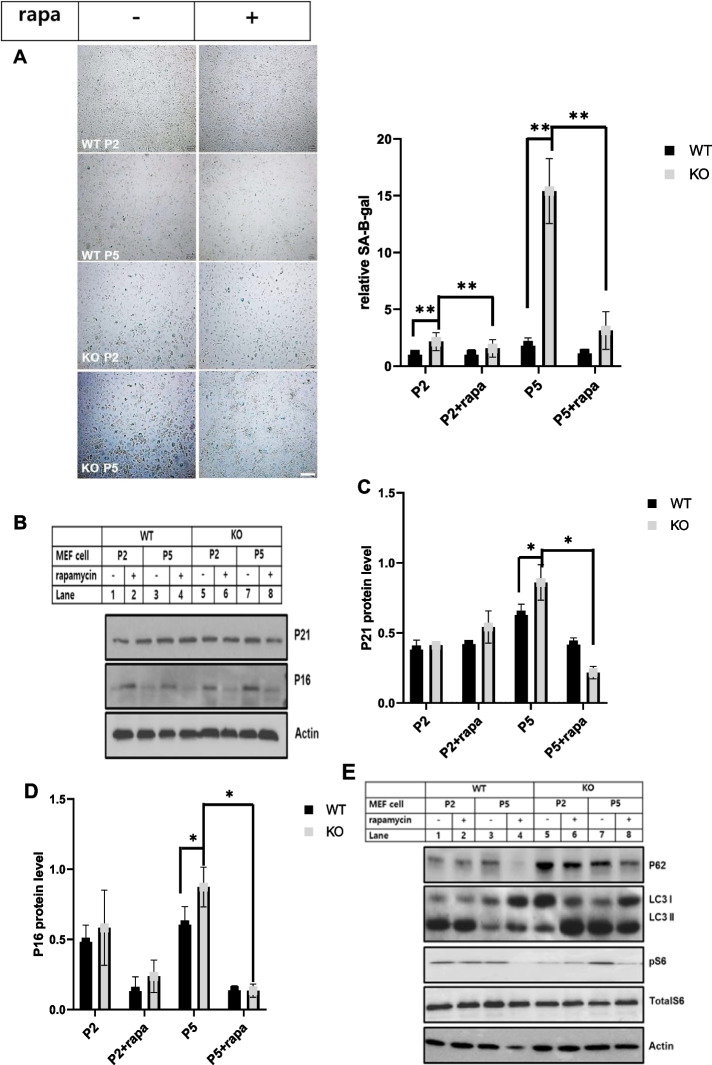

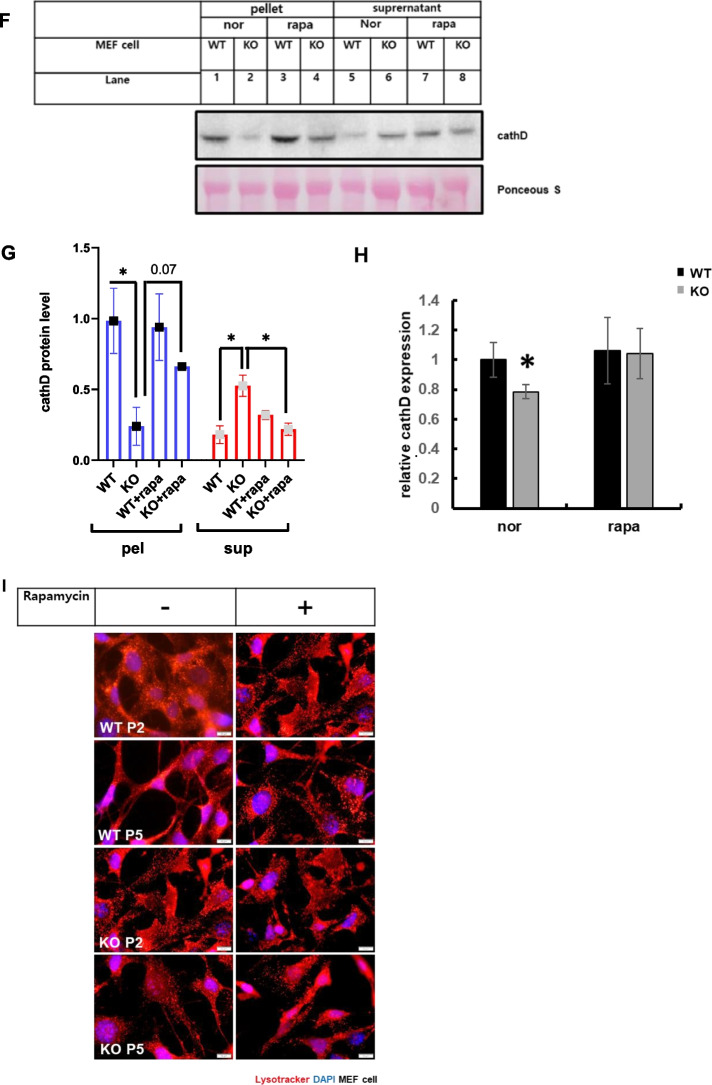
Rapamycin attenuated cellular senescence induced by catalase-deficient cell. **A** MEFs at P2 and P5 were treated with 1 μM rapamycin (rapa) overnight and senescence-associated β-galactosidase staining was analyzed. The intensities of senescent cells was analyzed in WT and KO MEFs treated with rapa. Positive intensities of β-galactosidase staining were measured using the ImageJ software. Bar graph represents mean ± SD (n = 3). Scale bar represents 200 μm. **B** Protein was extracted from MEFs as in A. Immunoblot analysis was performed using whole-cell lysates for senescence related antibodies. **C**, **D** Quantified protein level of P21and P16 normalized with actin. **E** Immunoblot analysis was performed using whole-cell lysates for autophagy and mTORC1-related antibodies. **F** Lysates of MEFs treated as in a were fractioned as explained in the Materials and Methods section. Equivalent volumes of each fraction were subjected to immunoblotting using cath D antibodies. **G** Quantified protein level of cathD on pellet and supernatant fractionations, normalized with ponceus S. **H** Relative cath D levels were measured from MEF lysates, treated as in a. Bar graph represents mean ± SD (n = 3 experiments). *P < 0.05, WT P5 versus KO P5. **I** Representative fluorescence image of MEFs treated as in a, immunostained with Lysotracker (red) and DAPI (blue). Scale bar represents 20 μm. Bar graph represents mean ± SD (n = 3 experiments). *P < 0.05; **P < 0.0001

mTOR is a key component of cellular metabolism that promotes cell growth and proliferation via nutrient sensing. In addition to cellular growth and proliferation, mTOR has also been associated as a lifespan regulator in mice [36–39]. The lifespan-enhancing effects of rapamycin  have been linked to mTORC1 inhibition [40]. Hence, we treated cells with rapamycin, an mTORC1 inhibitor, to reverse cellular senescence induced by catalase-KO mice. For this, WT and KO MEFs were treated with rapamycin, and β-galactosidase staining was performed to check the senescence phenotype. KO MEFs displayed flattened and enlarged senescence phenotypic morphology at early passage (P2) and showed increased senescence phenotypic morphology with increasing passage (P5) in addition to positive staining for senescence-associated β-galactosidase, but not in WT MEFs (Fig. 7A). However, treatment with rapamycin inhibited positive staining of senescence-associated β-galactosidase, but KO MEFs still displayed flattened and enlarged senescence phenotypic morphology. Further immunoblot analysis of MEFs showed the induction of senescence-related proteins,P21 and P16 in KO cells that was suppressed by co-treatment with rapamycin (Fig. 7B–D). mTORC1 is also known to suppress autophagy, and activation of autophagy by suppression of mTORC1 can slow age by clearing the accumulating old and dysfunctional organelles [40, 41]. Hence, to check the clearance of old and dysfunctional organelles by autophagy, rapamycin was used to treat MEFs, and immunoblot analysis was performed. Remarkably, the increased levels of autophagy substrate marker P62 and autophagosome marker LC3II were significantly decreased by rapamycin in KO MEFs at P5 level (Fig. 7E). Furthermore, the mTORC1 marker protein pS6 was decreased by rapamycin, suggesting that autophagy was initiated (Fig. 7E). In addition, accumulation of lysosomal content, such as cath D, in the supernatant fraction in KO MEFs at level P5 was slightly suppressed by rapamycin (Fig. 7F, G). Similarly, cath D activity was also recovered in KO MEF following treatment with rapamycin (Fig. 7H). Additionally, we immunostained MEFs with lysotracker to measure the acidic probe of lysosomes by rapamycin. As expected, the decreased acidic puncta of lysosomes were recovered by rapamycin in KO MEFs at P5 (Fig. 7I). Taken together, we showed that mTORC1 depletion by rapamycin slightly attenuated the progression of aging in catalase-KO mice. We also tried to show aging progression by hyperactivation of mTOR by the point mutation S2215Y, identified in the human cancer genome database [42]. We transfected the FLAG-tagged mutated form of mTOR with its WT plasmid in MEFs, followed by immunostaining with lysotracker (Additional file 1: Fig. S4). Consistent with the aged phenotype, the mutated form showed a diffuse form of lysotracker, whereas in WT cells, lysotracker puncta were quite distinctive. Together, these data showed that mTOR hyperactivation in catalase-KO mice may aggravate the aging phenotype faster than in WT mice.

## Discussion

Aging comprises various probabilities and is usually treated as a discrete variable rather than a continuous variable. Thus, most studies focus on three specific life phases: adult, mature adult or middle age, and old. In mice, adult (3–6 months) group is the reference for any age change, whether it is developmental, maturational, or senescent; mature adult or middle age (10–14 months) refers to a stage in which senescent changes can be detected in some, but not all, biomarkers of aging; and old age (18–24 months) refers to a stage in which senescent changes can be detected in almost all biomarkers [13, 14]. In this study, we used two groups of mice, viz.., younger (~ 2 months or 9 W) that were not affected by senescent and mature adult mice (12 months or 53 W) that are on the way to senescence. Although the 53 W mice breed normally, they exhibited the senescent phenotype faster in catalase-KO mice. In addition, during extensive culture passages, normal primary cells undergo malignant progression through cytostasis and contribute to cell senescence induction [43]. Hence, primary MEFs in a recent study showed a senescent-like phenotype only after P5 levels. Thus, to mimic aging phenotye as in mice, we mentioned MEFs for P2 as young cells and P5 as senescent cells.

Senescence refers to a cellular response characterized by a state of stable cell cycle arrest in which proliferating cells become resistant to growth but remain viable and metabolically active after prolonged time in culture [44]. However, senescence is not just a cell cycle arrest. When the cell cycle is arrested, an inappropriate growth‐promotion pathway, such as mTOR, converts arrest into senescence and mTOR inhibitor rapamycin decelerates cell proliferation but preserves the re-proliferative potential of inactive cells that are lost during senescence [45, 46]. Based on these findings, we concluded that rapamycin treatment decelerates cell proliferation but inhibits positive staining of senescence-associated β-galactosidase in KO aged MEFs (Fig. 7).

Lipofuscin is comprised of highly oxidized proteins, lipids, and metal elements that accumulate primarily in the lysosomes and reduce its autophagic capacity [47, 48]. These non-degradable lipids and ions or waste accumulation in the form of lipofuscin elevates the lysosomal pH, leading to a snowball effect on lysosomes [49]. Hence, the continuous accumulation of lipofuscin in lysosomes through ROS generation in catalase-KO mice increased the volume of lysosomes. Although lysosomal size increased, lysosomal hydrolytic activity decreased in catalase-KO aged mice (Fig. 3).

We showed catalase KO mature adult mice dysregulate autophagy through LMP. However by treatment with well-known LMP marker LLOME in hepG2 cell induce autophagy. We argued this discrepancies as LLOME is a lysosomotropic reagent that make lysosome damage through LMP (19). However, the cellular response mechanisms sense the damage followed by extensive modification of the organelles and mark them for clearance through autophagy and ensure lysosomal quality control and homeostasis [34]. Hence TFEB, a major regulator of autophagy and lysosomal biogenesis, phosphorylates and trafficked to nucleus for clearance of damaged lysosomes [35]. Although leaky lysosome was induced in catalase KO mature adult mice was due to the lipofuscin accumulation in lysosome that cause dysregulation of basal autophagy.

In contradicts to our study, it was shown that, catalase deficient mice shortens lifespan regardless of oxidative stress and premature aging [50]. Further, it revealed that catalase KO mice induced cellular senescence by β-galactosidase staining, but they failed to find any increasing level of senescence-related proteins, P21, P16 and P53 in the mice and concluded that catalase deficient mice shortens lifespan without premature aging [50]. This discrepancies from our study might be due to the genetic strains of mice from our study to other is different. We use from C57BL/6 strain, where as other use C57BL/6 N strain for study. Although both strain of mice are black, but those mice carry indiscriminate mutations and hence can affect the results of metabolic studies [51]. However, Perez-Estrada et al. agreed that catalase as a key regulator of longevity.

Although aging affects many cellular components, our study showed remarkable changes in lysosomes that induce mTORC hyperactivation and, thus, cause an aging phenotype faster in catalase-deficient mature adult mice. Our study may unveil the innovative roles of catalase and its relation to lysosomes and its role in aging.

## Conclusions

**Fig. 8:**
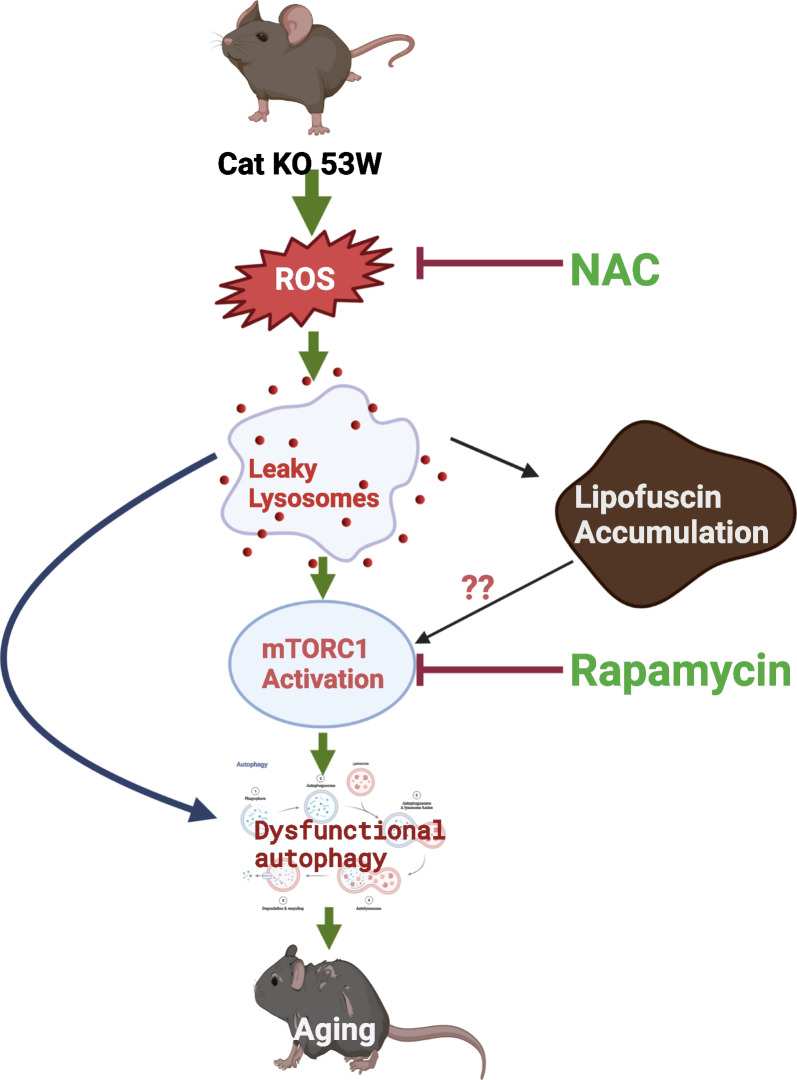
Graphical representation of catalase-deficient mice induce aging faster through lysosomal dysfunction

Our study clarified catalase as a key regulator for cellular senescence and aging. Depletion of catalase in mice induce aging faster through generation of ROS, that leads to leaky lysosome and hence basal autophagy is dysregulated through lipofuscin accumulation and hyper activation of mTORC1. That leads to generation of aging in mature adult mice (Fig. 8).

Hence catalase could be protective against the toxic hydrogen peroxide and might be the therapeutic interventions towards senescent cells that allow restoring the health span through detoxifying hydrogen peroxide.

## Supplementary Information

**Additional file 1: Fig. S1**. Catalase deficient MEF cells induce cellular senescence faster than its WT. **Fig. S2**. Catalase deficient aged mice slightly induced body weight. **Fig. S3**. Cellular senescence and MTORC1 is induced in LLOME treated HepG2 cells. **Fig. S4**. Hyperactivation of mTORC1 affects lysosomal acidity. **Table S1.** List of antibodies.

## Data Availability

All data generated and/or analyzed in this study are available from the corresponding author upon reasonable request.

## Abbreviations

9 W: 9 Weeks
53 W: 53 Weeks
P2: Passage 2
P5: Passage 5
LMP: Lysosomal membrane permeabilization
TFEB: Transcription factor EB
LLOME: L-leucyl-L-leucine methyl ester
