# Supplementary material for: Catalase-deficient mice induce aging faster through lysosomal dysfunction
Source: Cell Commun Signal. 2022 Dec 6;20:192. doi: 10.1186/s12964-022-00969-2 (PMC9724376; doi:10.1186/s12964-022-00969-2)
Supplement: Supplementary file 2 — Additional file 1: Fig. S1. Catalase deficient MEF cells induce cellular senescence faster than its WT. Fig. S2. Catalase deficient aged mice slightly induced body weight. Fig. S3. Cellular senescence and MTORC1 is induced in LLOME treated HepG2 cells. Fig. S4. Hyperactivation of mTORC1 affects lysosomal acidity. Table S1. List of antibodies. [file 12964_2022_969_MOESM2_ESM.docx]

**Supplementary information**

**Materials and methods**

Human wild-type pcDNA3-Flag mTOR WT (Plasmid #26603), activating MTOR mutation pcDNA3-FLAG-MTOR-S2215Y (Plasmid #69013) were obtained from Addgene. Plasmid transfection was performed with Lipofectamine 3000 reagent (Invitrogen) according to the manufacturer’s transfection protocol. Cells were fixed/harvested 12–24 h after transfection.

**S1. Catalase deficient MEF cells induce cellular senescence faster than its WT**


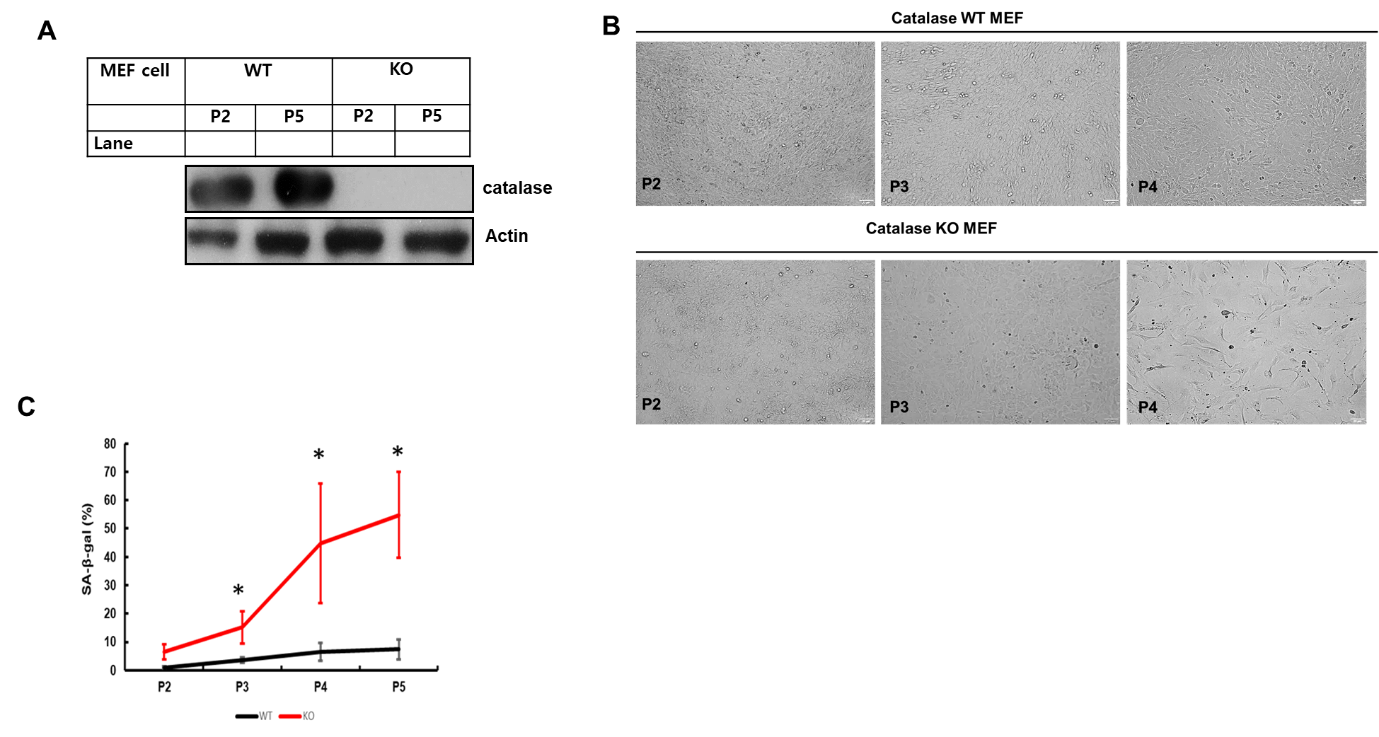


(a) Immunoblot were performed from MEFs from WT and catalase KO mice with indicated antibodies. (b) Morphology WT and catalase KO MEFs at passage (P2, P3 and P4) levels. (c) The bar graph representing the senescence-associated β-galactosidase staining of passage WT and catalase KO MEFs from P2 to P5 levels. Positive intensities of β-gal staining were measured using ImageJ software. mean ± SD (n=3 experiments). Scale bar represents 100 μm. *P < 0.05, WT P3 Vs KO P3; WT P4 Vs KO P4; WT P5 vs KO P5

**
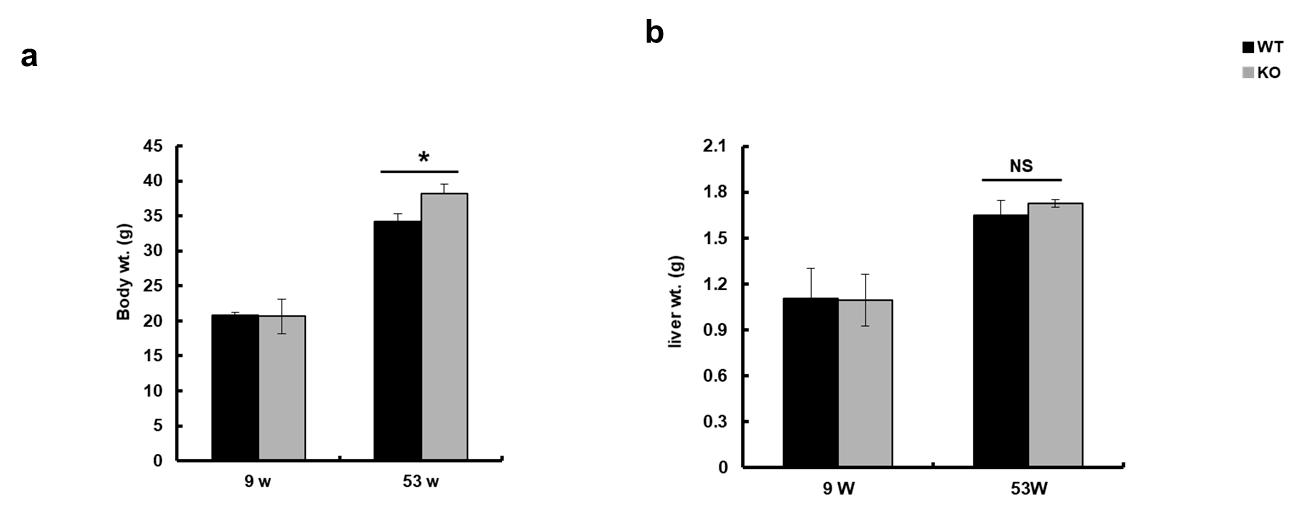
**

**S2. Catalase deficient aged mice slightly induced body weight**

(a) Body and (b) liver weight of WT and catalase KO mice at 9W and 53W in gram (g). The bar graph represents the mean ± SD (n=5 mice). *P < 0.05, WT 53W Vs KO 53W; ns: non-significant

**
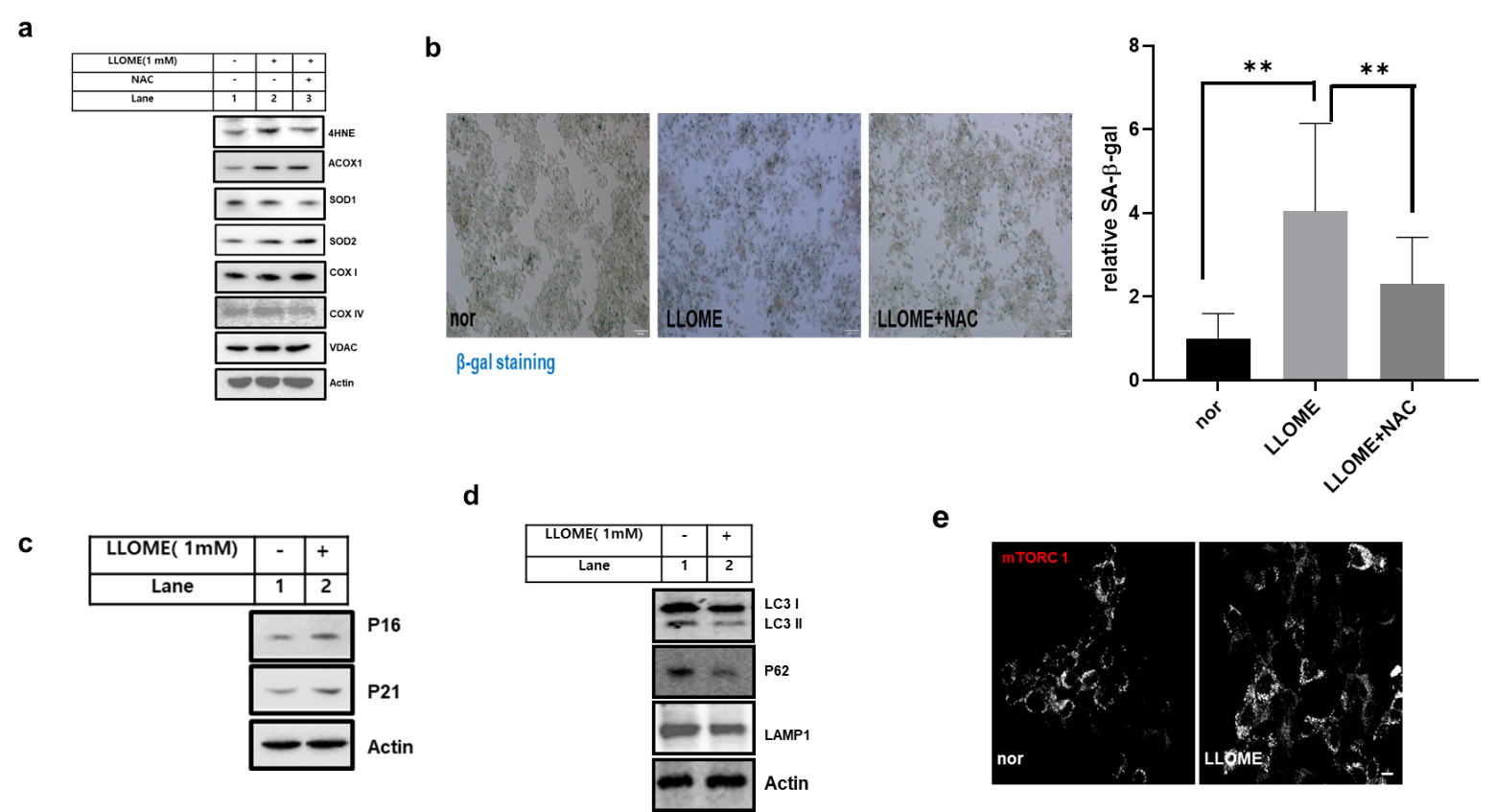
**

**S3. Cellular senescence and MTORC1 is induced in LLOME treated HepG2 cells**

1 mM of LLOMe were treated in HepG2 cell for 24 h and were co-treated with 5 mM of NAC for overnight. (a) Protein were extracted from HepG2 cells and immunoblot analysis was performed using whole-cell lysates with indicated antibodies. (b) senescence-associated β-galactosidase staining of HepG2 cells. **P < 0.001, (c-d) Protein were extracted from HepG2 cells and immunoblot analysis was performed using whole-cell lysates with indicated (c) senescence and (d) autophagy associated antibodies. (e) Representative fluorescence images HepG2 cells, fixed and immunostained with anti-mTORC1.

**
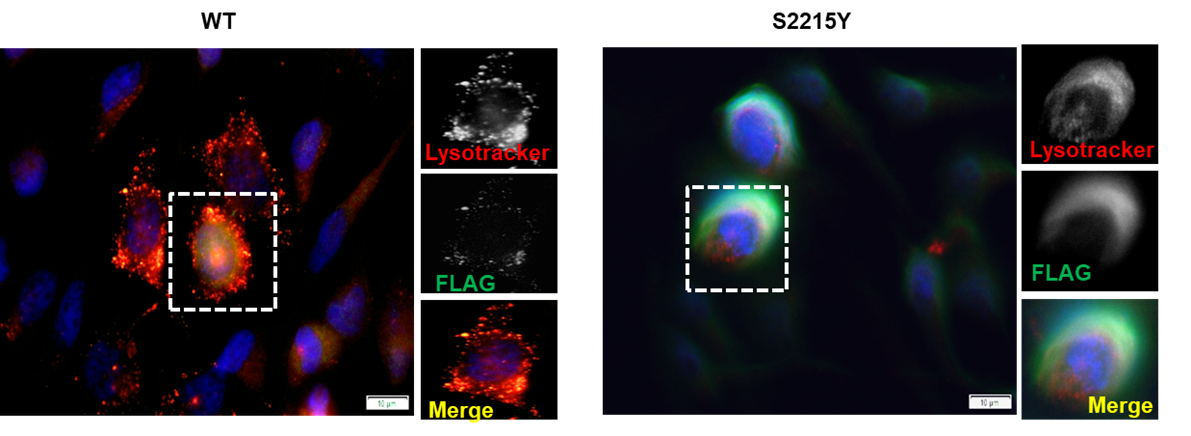
**

**S4. Hyperactivation of mTORC1 affects lysosomal acidity**

MEF cell were transfected with Human wild-type pcDNA3-Flag mTOR WT and/ or activating MTOR mutation pcDNA3-FLAG-MTOR-S2215Y for 24 h and were immunostained with lysotracker (Red), Flag (green) and Dapi( Blue). Scale bar, 10 μm.

**Table S1. List of antibodies used in this study**

| **antibody** | **Application** | **Dilution** | **company** | **Cat. No.** | **source** |
| --- | --- | --- | --- | --- | --- |
| ACOX1 | WB | 1:1000 | proteintech | 10957-1-AP | rabbit |
| p21 | WB | 1:1000 | Invitrogen | MA5-14949 | rabbit |
| catalase | WB | 1:1000 | abcam | ab4877 | rabbit |
| p16 | WB | 1:1000 | abcam | ab51243 | rabbit |
| Actin | WB | 1:1000 | Santa cruz | sc-47778HRP | - |
| 4-HNE | WB and IF | 1:1000 and 1:500 | abcam | ab-46545 | rabbit |
| LAMP1 | WB and IF | 1:1000 and 1:500 | abcam | ab24170 | rabbit |
| Cathespin D | WB | 1:1000 | invitrogen | MA5-17236 | mouse |
| Cathespin B | WB | 1:1000 | invitrogen | PA5-14255 | rabbit |
| LC3 | WB and IF | 1:1000 and 1:500 | Sigma -Aldrich | L8918 | rabbit |
| P62 | WB | 1:2000 | Abnova | H00008878-M01 | mouse |
| Galactin 1 | IF | 1:500 | abcam | ab-25138 | rabbit |
| TFEB | IF | 1:500 | MyBioScience | MBS120432 | mouse |
| Phospho-S6 | WB | 1:1000 | Cell Signalling | 2215S | rabbit |
| Total –S6 | WB | 1:1000 | Cell Signalling | 2217S | rabbit |
| SOD1 | WB | 1:1000 | Santa cruz | Sc-17767 | mouse |
| SOD2 | WB | 1:1000 | Santa cruz | Sc-30080 | rabbit |
| COX I | WB | 1:1000 | Santa cruz | sc-65237 | mouse |
| COX IV | WB | 1:1000 | invitrogen | 459600 | mouse |
| VDAC | WB | 1:1000 | calbiochem | 529532 | mouse |
| mTORC1 | IF | 1:500 | BioSource | AH01212 | rabbit |

IF: Immunofluorescence

WB: Western blot
